# Genetic predisposition to neurodegenerative diseases and risk of stroke: A Mendelian randomization study

**DOI:** 10.3389/fnins.2022.995045

**Published:** 2022-11-07

**Authors:** Fuju Li, Yunyun Wang, Xiaoyan Hou, Lina Cao, Xiaoyi Zhou, Weiyan Yuan, Yi Shen, Tian Tian

**Affiliations:** ^1^Department of Epidemiology and Health Statistics, School of Public Health, Nantong University, Nantong, China; ^2^Center for Disease Control and Prevention of Nantong, Nantong, China; ^3^Department of Gastroenterology, Affiliated Hospital of Nantong University, Nantong, China

**Keywords:** neurodegenerative diseases, stroke, Mendelian randomization, single nucleotide polymorphism, genome-wide association studies

## Abstract

**Background:**

Traditional epidemiological studies suggested that Neurodegenerative diseases (ND) might correlate with stroke. We intend to explore whether the two most common neurodegenerative diseases [Alzheimer’s disease (AD) and Parkinson’s disease (PD)] are causally associated with stroke and its subtypes.

**Methods:**

Two-sample Mendelian Randomization (MR) method was used to explore the causal relationships. Candidate genetic instrumental variables (IVs) for AD and PD were collected from the genome-wide association studies (GWAS) in European populations. The inverse-variance weighted (IVW) method was the primary method of MR analysis, and the weighted median method was supplementary. In addition, the MR-Egger method and the MR-PRESSO test were used as well.

**Results:**

We found no causal effects of AD on stroke, Ischemic stroke (IS), or Intracerebral hemorrhage (ICH). As for PD and stroke, our preliminary results showed PD could causally influence the risk of stroke [odds ratio (OR): 1.04; 95% confidence interval (CI): 1.02–1.07; *P* = 0.001 by the IVW method], although the alternative method did not support this result. We identified the positive causal relationship between PD and the risk of IS (OR = 1.04; 95% CI: 1.02–1.07; *P* = 0.001 by the IVW method), and the alternative MR methods produced similar results. The present study found there was no causal relationship between PD and ICH.

**Conclusion:**

This study found a causal relationship between genetic susceptibility to PD and the incidence of stroke (especially IS) in the European population; however, there was no causal relation between AD and stroke risk.

## Introduction

As the population of the world ages, the incidence of age-related diseases is increasing (Shlisky et al., 2017; Katzir et al., 2021). Stroke is one of the leading causes of death and disability worldwide, and the global burden of stroke continues to increase dramatically (Chong et al., 2019). In 2019, the number of deaths caused by stroke was 6.55 million, and the total number of stroke-induced disability-adjusted life years reached 143 million (Roth et al., 2020). Stroke could be classified as ischemic and hemorrhagic stroke, and ischemic stroke (IS) is the predominant type, which accounts for approximately 80% of all stroke patients (Rosamond et al., 2008; Rousselet et al., 2018). Intracerebral hemorrhage (ICH) is the most common type of hemorrhagic stroke (Montaño et al., 2021). Stroke is a complicated multi-factorial disease. Both environmental factors and genetic factors could increase the risk of stroke. The established environmental factors included tobacco smoking, physical inactivity, unhealthy diet, etc (Guzik and Bushnell, 2017). In the past decades, increasing evidence shows that genetic factors might play essential roles in the occurrence and development of stroke (Fu et al., 2019).

Neurodegenerative diseases (ND), with a high incidence in the elderly population as well, have seriously affected the life quality of older persons and imposed a heavy burden on the world (Meng et al., 2021). Alzheimer’s disease (AD) and Parkinson’s disease (PD) are the two most common age-related ND (Han et al., 2018). Previous studies have shown strong relationships between the above two age-related ND and stroke (Liu et al., 2020; Pinho et al., 2021). A meta-analysis in 2021 showed a significantly higher incidence of stroke in AD patients with an incidence rate ratio (IRR) of 1.31 (Pinho et al., 2021), indicating that AD might increase the risk of stroke. In 2020, a meta-analysis with 13 case-control studies identified a statistically significant association between PD and stroke (Liu et al., 2020). Besides, many scholars proposed that PD patients have an increased risk of stroke (Huang et al., 2019; Liu et al., 2020). Two nationwide population-based cohort studies demonstrated that PD was a vital independent risk factor for stroke (Huang et al., 2019). Nevertheless, these findings were merely produced by traditional epidemiological studies (including case-control studies and cohort studies) and might be influenced by the potential confounding factors or reverse causation bias.

An approach, mendelian randomization (MR), could overcome the above limitations of conventional epidemiologic studies (Georgakis et al., 2019). This approach uses genetic variants (single nucleotide polymorphisms, SNPs) to explore whether there is a causal effect of a suspicious influence factor on an outcome (Zuber et al., 2020). In the past few decades, MR has been widely used to assess causality (Zou et al., 2020). Some researchers even called MR the most valuable alternative to randomized controlled trials (RCTs) (Lawlor et al., 2008).

Thus, in this study, to systematically clarify the causal effects of the two age-related ND on stroke (including IS and ICH), we intended to use MR for further exploration, which might improve the prevention and treatment of stroke.

## Materials and methods

### Study design

**FIGURE 1:**
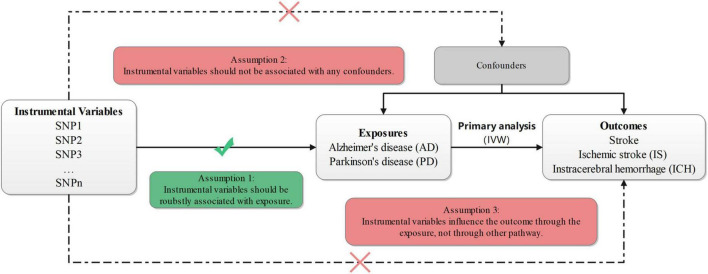
Design flow chart of the present study. IVW, inverse-variance-weighted; SNP, single-nucleotide polymorphism.

In this study, our two-sample MR intended to test whether the genetically instrumental variables (IVs) for AD or PD (the exposure factors) were also associated with stroke or different types of stroke (the outcomes). The IVs were independent SNPs for the exposures from Genome-Wide Association Studies (GWAS). As required for data analysis in the two-sample MR study, exposure- and outcome-relevant data were from non-overlapping data sources (Pierce and Burgess, 2013), and the details of related data were provided in Supplementary Table 1. Both samples (one for each exposure and another for each outcome) were from European populations. Owing to the use of summary-level data from GWAS, our MR study could overcome the limitations of small study populations, publication bias, etc., which might reduce the risk of false findings (Zhao et al., 2021). The study design flow chart is shown in Figure 1.

All data in this study were from public databases, and no additional ethical approval or consent to participate is needed.

### Selection of the genetic instrumental variables (exposure variables)

Candidate IVs for AD were collected from four consortia involving 21,982 cases and 41,944 controls, which were available on the MR-Base platform.^1^ The four consortia included Alzheimer Disease Genetics Consortium, Cohorts for Heart and Aging Research in Genomic Epidemiology Consortium, The European Alzheimer’s Disease Initiative, and Genetic and Environmental Risk in AD/Defining Genetic, Polygenic and Environmental Risk for Alzheimer’s Disease Consortium. A total of 21 independent SNPs associated with AD were extracted according to the GWAS threshold (*P* < 5 × 10^–8^) and linkage disequilibrium (LD) threshold (*r^2^* < 0.001) (Kunkle et al., 2019), and the relevant details were provided in Supplementary Table 2. The 21 SNPs were available for our further MR studies. For stroke and IS, 2 palindromic SNPs were removed from the relevant MR studies because they were palindromic with intermediate allele frequencies, leaving 19 SNPs. After data harmonization for AD and ICH, only 6 SNPs were selected as genetic instruments to examine the causal relation of AD to ICH.

PD genetic data were obtained from the International Parkinson’s Disease Genomics Consortium (37,688 cases, 18,618 UK Biobank proxy cases, and 1,417,791 controls). According to a study in 2019 (Nalls et al., 2019), 90 SNPs were independently associated with PD, all of which reached the genome-wide association threshold (*P* < 5 × 10^–8^), and the details are provided in Supplementary Table 3. After data harmonization for PD and stroke (including IS and ICH), 86 SNPs (4 SNPs of the 90 were deleted because of no proxies or palindromic structure) for stroke, 86 SNPs (4 SNPs of the 90 were eliminated because of no proxies or palindromic structure) for IS, and 59 SNPs (31 SNPs of the 90 were deleted because of no proxies or palindromic structure) for ICH were collected as genetic instruments.

### Data sources for the study outcome

In the present study, stroke-related genetic data came from the MEGASTROKE consortium. The detailed information about the consortium has been described in several published studies (Malik et al., 2018), and the details of MEGASTROKE consortium members were provided in the Supplementary material. In brief, the summary statistics were derived from 40,585 stroke cases and 406,111 controls of European ancestry. Genetic association data of IS came from 34,217 cases and 406,111 controls, available on the MR-Base platform (see text footnote 1). ICH-related genetic association data was obtained from a GWAS meta-analysis (Woo et al., 2014), including 1,545 cases and 1,481 controls, which were available at https://hugeamp.org/downloads.html.

### Statistical analysis

In our study, MR analysis was performed using four methods: inverse variance weighting (IVW), weighted median, MR-Egger, and Mendelian randomization pleiotropy residual sum and outlier (MR-PRESSO) method. The IVW was used as the primary method to estimate the causal effects of two age-related neurodegenerative diseases (AD and PD) on stroke and certain stroke subtypes (IS and ICH). The IVW method is to regress the instrument-outcome associations on the instrument-exposure associations, not considering the intercept term (Choi et al., 2020). And when the causal effect weight from the valid variants reached 50%, the weighted median method could provide consistent causal estimates (Bowden et al., 2016). MR-Egger method includes an intercept, which could explain the average pleiotropy effect of all genetic variations (Bowden et al., 2015). In addition, to further assess whether the presence of pleiotropic IVs influences on the causal estimation, we used the MR-PRESSO method to detect horizontal pleiotropy and correct it via outlier removal (Verbanck et al., 2018). Then, a sensitivity analysis was performed based on the PhenoScanner Platform.^2^ We removed the SNPs related to the secondary traits from the selected IVs, and performed the relevant MR analyses again.

All analyses were performed using R version 4.5.0.^3^ And the related R packages included MendelianRandomization (Hemani et al., 2018), MR-PRESSO (Verbanck et al., 2018), etc. Considering the issue of multiple testing in this study, after the Bonferroni corrections, the main results possessed statistical significance at *P*-values <0.017 (0.050/3). *P*-values from 0.017 to 0.050 could indicate that the causal relationships between exposures and outcomes are potential statistical significance (Xu et al., 2022).

## Results

### Instrumental variables in this study

To conduct MR analyses for AD and stroke (including IS and ICH), 19 SNPs for stroke, 19 SNPs for IS, and 6 SNPs for ICH were selected. As for PD and stroke (including IS and ICH), 86 SNPs for stroke, 86 SNPs for IS, and 59 SNPs for ICH were collected as genetic instruments for the MR analyses.

### Mendelian randomization analysis results for Alzheimer’s disease and stroke (including ischemic stroke and intracerebral hemorrhage)

#### Alzheimer’s disease and stroke

**FIGURE 2:**
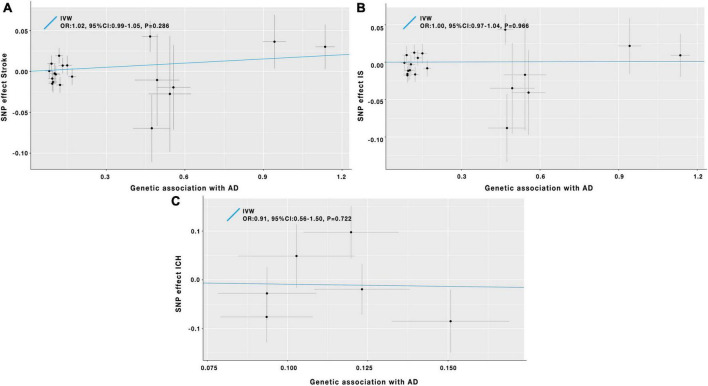
Scatter plot of the causal effects of AD on the risk of stroke **(A)**, IS **(B)**, and ICH **(C)**. IV, instrumental variable; IVW, inverse-variance-weighted; SNP, single-nucleotide polymorphism; OR, odds ratio; CI, confidence interval; AD, Alzheimer’s disease; IS, ischemic stroke; ICH, intracerebral hemorrhage.

As shown in Table 1 and Figure 2A, the IVW analysis results showed no causal relationship between genetically predicted AD and stroke risk (OR = 1.02; 95% CI: 0.99–1.05; *P* = 0.286). The weighted median method also yielded the similar result (OR = 1.05; 95% CI: 0.99–1.07; *P* = 0.140) in Table 1. According to the result of the MR-Egger analysis (*P*_for intercept_ = 0.200, in Table 1), there were no signs of directional pleiotropy. In addition, the MR-PRESSO method did not find any outlier SNPs or the pleiotropic effects of AD on stroke risk (*P* = 0.195) (Table 1). After manually searching on the PhenoScanner website, among the 19 SNPs, we found 8 potential pleiotropic SNPs, which were associated with AD-related secondary traits (Supplementary Table 4). Based on the left 11 SNPs, the results of MR analyses were similar to the previously mentioned results, and the details are shown in Supplementary Table 6.

**TABLE 1:** Mendelian randomization analysis results for the causal associations of two common neurodegenerative diseases with stroke.

Method	Alzheimer’s disease				Parkinson’s disease			
	SNPs (N)	OR	95% CI	*P*-value	SNPs (N)	OR	95% CI	*P*-value
**Stroke**								
IVW	19	1.02	0.99∼1.05	0.286	86	**1.04**	**1.02∼1.07**	**0.001**
Weighted median	19	1.05	0.99∼1.07	0.140	86	**1.04**	**1.00∼1.08**	**0.051**
MR Egger	19	–	–	0.200^a^	86	–	–	0.144^a^
MR-PRESSO	19	–	–	0.195^b^	86	–	–	0.835^b^
**Ischemic stroke**								
IVW	19	1.00	0.97∼1.04	0.966	86	**1.04**	**1.02∼1.07**	**0.001**
Weighted median	19	1.01	0.97∼1.06	0.606	86	**1.05**	**1.00∼1.09**	**0.040**
MR Egger	19	–	–	0.175^a^	86	–	–	0.158^a^
MR-PRESSO	19	–	–	0.207^b^	86	–	–	0.655^b^
**Intracerebral hemorrhage**								
IVW	6	0.91	0.56∼1.50	0.722	59	0.96	0.80∼1.16	0.688
Weighted median	6	0.80	0.47∼1.36	0.409	59	0.97	0.74∼1.27	0.811
MR Egger	6	–	–	0.966^a^	59	–	–	0.578^a^
MR-PRESSO	6	–	–	0.192^b^	59	–	–	0.666^b^

SNP, single nucleotide polymorphism; OR, odds ratio; CI, confidence interval; IVW, inverse-variance-weighted; MR, Mendelian randomization; MR-PRESSO, MR pleiotropy residual sum and outlier.

^a^*P*-value of the intercept from MR Egger regression analysis.

^b^*P*-value of MR-PRESSO global test.

Bold values represent the relevant results that were statistically significant or marginally statistically significant.

#### Alzheimer’s disease and ischemic stroke

Results of the IVW analysis indicated no causal association between AD and IS (OR = 1.00; 95% CI: 0.97–1.04; *P* = 0.966) in Table 1 and Figure 2B. Similar results were obtained by the weighted median analysis (OR = 1.01; 95% CI: 0.97–1.06; *P* = 0.606) in Table 1. The MR-Egger analysis did not detect any evidence of pleiotropy (*P*_for intercept_ = 0.175, in Table 1). As shown in Table 1, the MR-PRESSO method also did not find outlier SNPs (*P* = 0.207). After removing the 8 SNPs associated with AD-related secondary phenotypes (Supplementary Table 4), the MR analysis results based on the remained 11 SNPs were similar to the MR results mentioned above (Supplementary Table 6).

#### Alzheimer’s disease and intracerebral hemorrhage

In Table 1 and Figure 2C, the results of the IVW method proved that AD was not causally associated with ICH (OR = 0.91; 95% CI: 0.56–1.50; *P* = 0.722). The other three MR analysis methods provided similar findings in Table 1. Besides, the MR-Egger method showed no signs of directional pleiotropy. After removing the pleiotropic SNPs (Supplementary Table 4), the MR analysis results on the left 4 SNPs showed no causal relationship between AD and ICH (Supplementary Table 6).

### Mendelian randomization analysis results for Parkinson’s disease and stroke (including ischemic stroke and intracerebral hemorrhage)

#### Parkinson’s disease and stroke

**FIGURE 3:**
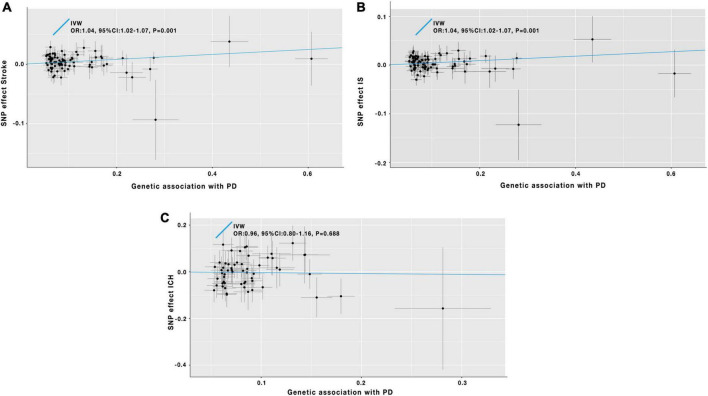
Scatter plot of the causal effects of PD on the risk of stroke **(A)**, IS **(B)**, and ICH **(C)**. IV, instrumental variable; IVW, inverse-variance-weighted; SNP, single-nucleotide polymorphism; OR, odds ratio; CI, confidence interval; PD, Parkinson’s disease; IS, ischemic stroke; ICH, intracerebral hemorrhage.

As the IVW analysis results showed in Table 1 and Figure 3A, a positive causal effect of PD on stroke was identified (OR = 1.04; 95% CI: 1.02–1.07; *P* = 0.001). The weighted median analysis suggested the above causal relationship reached the borderline statistical significance (OR = 1.04; 95% CI: 1.00–1.08; *P* = 0.051) in Table 1. The MR-Egger method detected no directional pleiotropy for instrumental variables (*P*_*for intercept*_ = 0.144, in Table 1). No SNP outliers were identified by the MR-PRESSO method (*P* = 0.835, in Table 1). Manually searching on the PhenoScanner website helped us to identify 26 potential pleiotropic SNPs (Supplementary Table 5). After exclusion of these SNPs, based on the left 60 SNPs, the MR effect estimate of PD on stroke risk did not change significantly (Supplementary Table 5). However, the weighted median analysis could not prove the causal relationship between PD on stroke risk (Supplementary Table 6).

#### Parkinson’s disease and ischemic stroke

The IVW analysis results in Table 1 and Figure 3B indicated that PD was genetically associated with an increased IS risk (OR = 1.04; 95% CI: 1.02–1.07; *P* = 0.001). A similar significant association was also obtained using the weighted median model (OR = 1.05; 95% CI: 1.00–1.09; *P* = 0.040) in Table 1. No pleiotropy was discovered by the MR-Egger regression method (*P*_for intercept_ = 0.158, in Table 1). The MR-PRESSO method did not detect outlier SNPs in our study (*P* = 0.655, in Table 1). After manually searching on the PhenoScanner website, 26 SNPs with pleiotropy were excluded (Supplementary Table 5). The remaining 60 SNPs were used for the secondary MR analyses. And the relevant results were consistent with our previous findings of the initial MR analyses (Supplementary Table 6).

#### Parkinson’s disease and intracerebral hemorrhage

In Table 1 and Figure 3C, the IVW analysis results showed no genetically causal relationship between PD and ICH risk (OR = 0.96; 95% CI: 0.80–1.16; *P* = 0.688). And the findings of other MR-analysis methods still showed no causal association between PD and ICH risk (Table 1). After deleting the potential pleiotropic SNPs (Supplementary Table 5), we conducted the MR analyses, and the results still showed no causal relationship between PD and ICH (Supplementary Table 6).

## Discussion

The present study systematically explored the causal relationships of AD and PD with the risk of stroke (including stroke subtypes), which enriched and improved the existing relevant findings. The analysis results showed no causal effects of AD on stroke, IS, or ICH. As for PD and stroke, we identified the positive causal relationship between PD and the risk of stroke and IS; however, the present study did not find evidence of the causal relationship between PD and ICH.

AD and stroke are two prevalent neurological diseases among elderly populations (Vijayan et al., 2017; Wang L. et al., 2020; Wang T. et al., 2020). And several observational studies showed there might be potential causal relationships between AD and stroke (Pinho et al., 2021). In order to explore the causal relationships between stroke and AD, Wang et al. conducted a MR study. They pointed out that different types of stroke would not be causally associated with AD risk (Wang T. et al., 2020). They also mentioned the reverse MR with the results indicating AD was not causally linked to stroke. However, they did not provide the reverse MR results in detail and did not explore the causal effects of AD on stroke subtypes. Our present results further approved that AD had no causal relationship with the risk of stroke. And our findings indicated that AD could not be significantly correlated with the development of stroke subtypes (IS or ICH). As Wang T. et al. (2020) discussed, AD and stroke are two complex neurological diseases, and their development and progression would be caused by other factors, which might need more effort to explore in the future.

PD has been approved to be obviously associated with stroke (Alves et al., 2020; Liu et al., 2020). Huang et al. performed a follow-up study and proposed that there was a significantly increased risk of IS in PD patients (Huang et al., 2013). So far, two MR studies have suggested that PD is causally affected by the risk of IS in European populations (Fang et al., 2022; Wang et al., 2022). Similar to their findings, our present study also identified the causal effects of PD on the risk of IS. Moreover, our present study discovered the causal relationship between PD and stroke and explored the potential genetic effect of PD on the risk of ICH, which has not been reported before. Besides, by comparison with Mengmeng Wang’s article (Wang et al., 2022), we added another sensitivity analysis to this study. After removing the SNPs associated with PD-related secondary phenotypes based on the PhenoScanner website, the secondary MR-analysis’s results repeated our initial ones, making our findings more credible.

The explicit mechanisms underlying the causal relationship between PD and stroke (especially IS) are still unclear. Hyperhomocysteinemia (elevated homocysteine levels) might potentially affect the above causal relationships (Becker et al., 2010; Supplementary Figure 1). Several studies have reported that PD patients exhibited higher plasma homocysteine levels than normal individuals (Ientile et al., 2010). Some epidemiological studies have identified that homocysteine was an independent risk factor for stroke (Kelly and Furie, 2002; Moretti and Caruso, 2019). Therefore, it is possible that the high level of plasma homocysteine of PD patients could increase their risk of developing stroke. Another potential mechanism is about blood pressure dysregulation, such as orthostatic hypotension, which is a symptom of many PD patients (Pfeiffer, 2016; Supplementary Figure 1). Orthostatic hypotension has been shown to increase the risk of stroke (Eigenbrodt et al., 2000; Mehta et al., 2019). Wang et al. proposed that atrial fibrillation might be another potential mechanism of PD on the risk of stroke (Wang et al., 2022; Supplementary Figure 1). Although the above explanations are biologically plausible, more well-designed explorations are still needed to confirm the exact mechanisms of the causal effect of PD on stroke risk.

Our present two-sample MR study overcame the shortcomings of the traditional epidemiological studies, including overcoming the effects of confounding and reverse causation (Smith and Ebrahim, 2003). The second strength was using various MR analysis methods for more exact results. Last but not least, the instrumental variables were selected based on relevant GWAS studies with larger sample sizes, which could maximize the statistical power. However, this study still had some limitations. All MR analyses of this study were limited to the European population, and the findings might not be suitable for other populations. Because our two-sample MR study was based on different studies, inevitable heterogeneity among studies might exist. However, the sensitivity analyses did not identify substantial pleiotropy in our study.

## Conclusion

Our study showed a causal effect of PD on the risk of stroke, especially IS; however, there was no causal relation between AD and stroke risk. Thus, we might need to pay more attention to PD patients to reduce their risk of developing stroke. In addition, more well-designed studies are required to explore the underline biological mechanisms.

## Data availability statement

The original contributions presented in this study are included in the article/Supplementary material, further inquiries can be directed to the corresponding authors.

## Author contributions

TT, YS, and WY designed the research. FL, YW, and XH had full access to all the data in the study and took responsibility for the integrity of the data and the accuracy of the data analysis, wrote the manuscript, and performed the data analysis. All authors contributed to the statistical analysis, critically reviewed the manuscript during the writing process, and approved the final version to be published.

## Footnotes

1
https://gwas.mrcieu.ac.uk/

2
http://www.phenoscanner.medschl.cam.ac.uk/

3
http://www.r-project.org
