# Supplementary material for: Genetic predisposition to neurodegenerative diseases and risk of stroke: A Mendelian randomization study
Source: Front Neurosci. 2022 Nov 7;16:995045. doi: 10.3389/fnins.2022.995045 (PMC9676950; doi:10.3389/fnins.2022.995045)
Supplement: Supplementary file 1 [file Data_Sheet_1.docx]

***Supplementary Material***

**Table S1.** Data information used in this study

| **Phenotype** | | **Sample Size** | **Ancestry** |
| --- | --- | --- | --- |
| Exposures | Alzheimer's disease(AD) | 21,982cases/41,944controls | European ancestry |
|  | Parkinson’s Disease (PD) | 56,306 cases^a^/ 1,417,791 controls | European ancestry |
| Outcomes | Stroke | 40,585 cases/406,111 controls | European ancestry |
|  | Ischemic stroke (IS) | 34,217 cases/406,111 controls | European ancestry |
|  | Intracerebral hemorrhage(ICH) | 1545cases/1481controls | European ancestry |

^a^37,688 cases, 18,618 UK Biobank proxy cases

**Table S2.** The characteristic information of instrumental variables for Alzheimer's disease in MR analyses

| SNP | Chr | Position | Gene | EA | OA | Beta | SE | P-value |
| --- | --- | --- | --- | --- | --- | --- | --- | --- |
| rs1081105 | 19 | 45412955 | APOC1 | C | A | 0.942 | 0.044 | 1.51E-103 |
| rs111278137 | 19 | 45215081 | CEACAM16 | A | G | -0.474 | 0.071 | 3.20E-11 |
| rs11257242 | 10 | 11721119 | RP11-138I18.2 | G | C | 0.084 | 0.015 | 4.64E-08 |
| rs114812713 | 6 | 41034000 | OARD1 | C | G | 0.298 | 0.043 | 4.47E-12 |
| rs11767557 | 7 | 143109139 | EPHA1 | C | T | -0.103 | 0.018 | 1.56E-08 |
| rs12151021 | 19 | 1050874 | ABCA7 | G | A | -0.107 | 0.017 | 2.56E-10 |
| rs12590654 | 14 | 92938855 | SLC24A4 | A | G | -0.091 | 0.016 | 8.73E-09 |
| rs139136389 | 19 | 45427136 | APOC1 | T | C | -0.494 | 0.085 | 6.43E-09 |
| rs147711004 | 19 | 45337918 | NECTIN2 | A | G | 1.135 | 0.037 | 1.00E-200 |
| rs150685845 | 19 | 45675180 | TRAPPC6A | G | A | 0.556 | 0.065 | 6.62E-18 |
| rs1582763 | 11 | 60021948 | MS4A4E | A | G | -0.123 | 0.015 | 1.19E-16 |
| rs34665982 | 6 | 32560306 | HLA-DRB1 | C | T | -0.097 | 0.017 | 5.80E-09 |
| rs3740688 | 11 | 47380340 | SPI1h | T | G | 0.094 | 0.014 | 9.70E-11 |
| rs3851179 | 11 | 85868640 | PICALM | C | T | 0.12 | 0.015 | 5.81E-16 |
| rs6733839 | 2 | 127892810 | BIN1 | T | C | 0.169 | 0.015 | 4.02E-28 |
| rs679515 | 1 | 207750568 | CR1 | C | T | -0.151 | 0.018 | 1.55E-16 |
| rs72654445 | 19 | 45417200 | APOC1 | A | G | -0.543 | 0.081 | 2.27E-11 |
| rs73223431 | 8 | 27219987 | PTK2B | T | C | 0.094 | 0.015 | 8.34E-10 |
| rs7412 | 19 | 45412079 | APOE | T | C | -0.467 | 0.031 | 6.40E-53 |
| rs867230 | 8 | 27468503 | CLU | A | C | 0.133 | 0.016 | 3.49E-17 |
| rs9381563 | 6 | 47432637 | AL355353.1 | T | C | -0.082 | 0.015 | 2.93E-08 |

MR, Mendelian randomization; SNP, single nucleotide polymorphism; Chr, chromosome; EA, effect allele; OA,other allele, SE, standard error.

**Table S3.** The characteristic information of instrumental variables for Parkinson's Disease in MR analyses

| SNP | Chr | Position | Gene | EA | OA | Beta | SE | P-value |
| --- | --- | --- | --- | --- | --- | --- | --- | --- |
| rs10221156 | 16 | 52969426 | CHD9 | A | G | -0.116 | 0.018 | 1.08E-10 |
| rs10513789 | 3 | 182760073 | MCCC1 | T | G | 0.149 | 0.012 | 1.22E-34 |
| rs10748818 | 10 | 104015279 | GBF1 | A | G | -0.079 | 0.013 | 1.05E-09 |
| rs10756907 | 9 | 17727065 | SH3GL2 | A | G | -0.093 | 0.011 | 5.06E-17 |
| rs10797576 | 1 | 232664611 | SIPA1L2 | T | C | 0.111 | 0.013 | 6.84E-17 |
| rs10847864 | 12 | 123326598 | HIP1R | T | G | 0.148 | 0.012 | 1.47E-37 |
| rs11150601 | 16 | 30977799 | SETD1A | A | G | 0.091 | 0.01 | 5.12E-20 |
| rs11158026 | 14 | 55348869 | GCH1 | T | C | -0.084 | 0.01 | 1.66E-16 |
| rs112485576 | 6 | 32578772 | HLA-DRB5 | A | C | -0.168 | 0.015 | 6.96E-28 |
| rs114138760 | 1 | 154898185 | PMVK | C | G | 0.281 | 0.048 | 4.19E-09 |
| rs11557080 | 1 | 205737739 | RAB29 | A | G | 0.132 | 0.014 | 2.50E-22 |
| rs11578699 | 1 | 171719769 | VAMP4 | T | C | -0.07 | 0.012 | 4.47E-09 |
| rs11610045 | 12 | 133063768 | FBRSL1 | A | G | 0.06 | 0.009 | 1.77E-10 |
| rs11658976 | 17 | 44866805 | WNT3 | A | G | -0.062 | 0.011 | 3.52E-08 |
| rs11683001 | 2 | 102396963 | MAP4K4 | A | T | 0.071 | 0.01 | 8.04E-13 |
| rs11707416 | 3 | 151108965 | MED12L | A | T | -0.063 | 0.01 | 1.13E-10 |
| rs117615688 | 17 | 43798308 | CRHR1 | A | G | -0.232 | 0.029 | 6.71E-16 |
| rs117896735 | 10 | 121536327 | INPP5F | A | G | 0.435 | 0.039 | 2.36E-28 |
| rs11950533 | 5 | 134199105 | C5orf24 | A | C | -0.092 | 0.016 | 7.16E-09 |
| rs12147950 | 14 | 37989270 | MIPOL1 | T | C | -0.053 | 0.01 | 3.54E-08 |
| rs12283611 | 11 | 83487277 | DLG2 | A | C | -0.065 | 0.01 | 2.61E-10 |
| rs12456492 | 18 | 40673380 | RIT2 | A | G | -0.098 | 0.01 | 3.80E-23 |
| rs12497850 | 3 | 48748989 | IP6K2 | T | G | 0.064 | 0.01 | 1.36E-10 |
| rs12528068 | 6 | 72487762 | RIMS1 | T | C | 0.066 | 0.01 | 1.63E-10 |
| rs12600861 | 17 | 7355621 | CHRNB1 | A | C | -0.057 | 0.01 | 1.01E-08 |
| rs1293298 | 8 | 11712443 | CTSB | A | C | 0.093 | 0.011 | 3.99E-16 |
| rs12951632 | 17 | 40741013 | RETREG3 | T | C | 0.064 | 0.011 | 1.40E-09 |
| rs13117519 | 4 | 114369065 | CAMK2D | T | C | 0.088 | 0.012 | 9.82E-13 |
| rs13294100 | 9 | 17579690 | SH3GL2 | T | G | -0.086 | 0.01 | 8.72E-18 |
| rs1450522 | 3 | 161077630 | SPTSSB | A | G | -0.062 | 0.01 | 5.01E-10 |
| rs1474055 | 2 | 169110394 | STK39 | T | C | 0.18 | 0.014 | 2.54E-39 |
| rs1867598 | 5 | 60137959 | ELOVL7 | A | G | -0.155 | 0.016 | 2.52E-23 |
| rs1941685 | 18 | 31304318 | ASXL3 | T | G | 0.053 | 0.009 | 1.69E-08 |
| rs199351 | 7 | 23300049 | GPNMB | A | C | 0.102 | 0.01 | 5.25E-26 |
| rs2042477 | 2 | 96000943 | KCNIP3 | A | T | -0.066 | 0.012 | 1.38E-08 |
| rs2086641 | 8 | 130901909 | FAM49B | T | C | -0.061 | 0.011 | 1.81E-08 |
| rs2248244 | 21 | 38852361 | DYRK1A | A | G | 0.071 | 0.011 | 2.74E-11 |
| rs2251086 | 15 | 61997385 | VPS13C | T | C | -0.119 | 0.014 | 6.08E-18 |
| rs2269906 | 17 | 42294337 | UBTF | A | C | 0.063 | 0.01 | 6.24E-10 |
| rs2280104 | 8 | 22525980 | BIN3 | T | C | 0.056 | 0.01 | 1.16E-08 |
| rs26431 | 5 | 102365794 | PAM | C | G | 0.062 | 0.01 | 1.57E-09 |
| rs2904880 | 16 | 28944396 | CD19 | C | G | -0.065 | 0.011 | 7.87E-10 |
| rs3104783 | 16 | 52636242 | CASC16 | A | C | 0.067 | 0.009 | 1.29E-12 |
| rs34025766 | 4 | 17968811 | LCORL | A | T | -0.084 | 0.013 | 2.87E-10 |
| rs34311866 | 4 | 951947 | TMEM175 | T | C | -0.213 | 0.012 | 9.98E-70 |
| rs34637584 | 12 | 40734202 | LRRK2 | A | G | 2.429 | 0.094 | 3.61E-148 |
| rs356182 | 4 | 90626111 | SNCA | A | G | -0.277 | 0.011 | 3.89E-154 |
| rs35749011 | 1 | 155135036 | KRTCAP2 | A | G | 0.607 | 0.034 | 1.72E-70 |
| rs3742785 | 14 | 75373034 | RPS6KL1 | A | C | 0.071 | 0.012 | 1.92E-09 |
| rs3802920 | 11 | 133787001 | IGSF9B | T | G | 0.107 | 0.012 | 6.26E-20 |
| rs4101061 | 4 | 77147969 | FAM47E | A | G | -0.091 | 0.01 | 4.97E-19 |
| rs4140646 | 6 | 27738801 | LOC100131289 | A | G | 0.083 | 0.012 | 5.62E-12 |
| rs4653767 | 1 | 226916078 | ITPKB | T | C | 0.083 | 0.01 | 1.38E-15 |
| rs4698412 | 4 | 15737348 | BST1 | A | G | 0.104 | 0.009 | 2.06E-28 |
| rs4771268 | 13 | 97865021 | MBNL2 | T | C | 0.068 | 0.011 | 1.45E-09 |
| rs5019538 | 4 | 90636630 | SNCA | A | G | -0.157 | 0.012 | 1.13E-36 |
| rs55818311 | 19 | 2341047 | SPPL2B | T | C | -0.07 | 0.011 | 4.18E-10 |
| rs55961674 | 3 | 122196892 | KPNA1 | T | C | 0.086 | 0.013 | 9.98E-12 |
| rs57891859 | 2 | 135464616 | TMEM163 | A | G | 0.081 | 0.011 | 4.55E-14 |
| rs61169879 | 17 | 59917366 | BRIP1 | T | C | 0.082 | 0.013 | 9.28E-10 |
| rs620513 | 8 | 16697593 | FGF20 | T | G | -0.086 | 0.011 | 2.72E-15 |
| rs62053943 | 17 | 43744203 | CRHR1 | T | C | -0.27 | 0.016 | 3.58E-68 |
| rs62333164 | 4 | 170583157 | CLCN3 | A | G | -0.064 | 0.01 | 2.00E-10 |
| rs6476434 | 9 | 34046391 | UBAP2 | T | C | -0.062 | 0.011 | 6.58E-09 |
| rs6497339 | 16 | 19277493 | SYT17 | A | T | 0.063 | 0.01 | 2.76E-11 |
| rs6500328 | 16 | 50736656 | NOD2 | A | G | 0.059 | 0.01 | 1.82E-09 |
| rs6658353 | 1 | 161469054 | FCGR2A | C | G | 0.065 | 0.009 | 6.10E-12 |
| rs666463 | 17 | 76425480 | DNAH17 | A | T | 0.076 | 0.013 | 3.20E-09 |
| rs6808178 | 3 | 28705690 | LINC00693 | T | C | 0.066 | 0.01 | 8.09E-12 |
| rs6825004 | 4 | 77110365 | SCARB2 | C | G | 0.062 | 0.01 | 1.17E-09 |
| rs6854006 | 4 | 77198054 | FAM47E-STBD1 | T | C | -0.091 | 0.01 | 5.82E-21 |
| rs7134559 | 12 | 46419086 | SCAF11 | T | C | -0.054 | 0.01 | 3.96E-08 |
| rs72840788 | 10 | 121415685 | BAG3 | A | G | 0.076 | 0.011 | 1.57E-11 |
| rs73038319 | 3 | 18361759 | SATB1 | A | C | -0.169 | 0.024 | 5.94E-13 |
| rs75859381 | 6 | 133210361 | RPS12 | T | C | -0.221 | 0.034 | 1.04E-10 |
| rs76116224 | 2 | 18147848 | KCNS3 | A | T | 0.11 | 0.019 | 1.27E-08 |
| rs76763715 | 1 | 155205634 | GBAP1 | T | C | -0.747 | 0.077 | 1.59E-22 |
| rs76904798 | 12 | 40614434 | LRRK2 | T | C | 0.144 | 0.013 | 1.52E-28 |
| rs76949143 | 7 | 66009851 | GS1-124K5.11 | A | T | -0.143 | 0.025 | 1.43E-08 |
| rs77351827 | 20 | 6006041 | CRLS1 | T | C | 0.08 | 0.014 | 8.87E-09 |
| rs7938782 | 11 | 10558777 | RNF141 | A | G | 0.087 | 0.015 | 2.12E-09 |
| rs8087969 | 18 | 48683589 | MEX3C | T | G | -0.058 | 0.01 | 1.41E-08 |
| rs823118 | 1 | 205723572 | NUCKS1 | T | C | 0.107 | 0.009 | 1.11E-29 |
| rs850738 | 17 | 42434630 | FAM171A2 | A | G | -0.071 | 0.011 | 1.29E-11 |
| rs873786 | 4 | 925376 | GAK | T | C | -0.173 | 0.018 | 1.79E-21 |
| rs896435 | 10 | 15557406 | ITGA8 | T | C | 0.074 | 0.01 | 3.41E-13 |
| rs9261484 | 6 | 30108683 | TRIM40 | T | C | -0.064 | 0.011 | 1.62E-08 |
| rs9568188 | 13 | 49927732 | CAB39L | T | C | 0.062 | 0.011 | 1.15E-08 |
| rs979812 | 14 | 88464264 | GALC | T | G | 0.061 | 0.009 | 6.19E-11 |
| rs997368 | 6 | 112243291 | FYN | A | G | 0.071 | 0.012 | 1.84E-09 |

SNP, single nucleotide polymorphism; Chr, chromosome; EA, effect allele; OA, other allele; SE, standard error.

**Table S4.** Associations between Alzheimer's disease loci and other traits (from the PhenoScanner)

| SNP | Trait | PMID | *P* |
| --- | --- | --- | --- |
| rs34665982 | Hemoglobin concentration | 27863252 | 1.12E-17 |
|  | Lymphocyte count | 27863252 | 1.91E-14 |
|  | Inflammatory bowel disease | 26192919 | 3.22E-14 |
|  | Reticulocyte count | 27863252 | 3.90E-14 |
|  | Ulcerative colitis | 26192919 | 1.79E-13 |
|  | Mean corpuscular hemoglobin concentration | 27863252 | 1.30E-11 |
|  | Reticulocyte fraction of red cells | 27863252 | 4.72E-11 |
|  | High light scatter reticulocyte count | 27863252 | 2.06E-10 |
|  | Hematocrit | 27863252 | 6.98E-10 |
|  | High light scatter percentage of red cells | 27863252 | 2.42E-08 |
| rs9381563 | Mean platelet volume | 27863252 | 4.73E-14 |
|  | Immature fraction of reticulocytes | 27863252 | 5.47E-12 |
|  | High light scatter reticulocyte count | 27863252 | 8.63E-11 |
|  | Platelet count | 27863252 | 1.48E-10 |
|  | High light scatter percentage of red cells | 27863252 | 7.79E-10 |
|  | Platelet distribution width | 27863252 | 2.91E-09 |
|  | Red cell distribution width | 27863252 | 1.39E-08 |
| rs1582763 | Heel bone mineral density | UKBB | 5.20E-13 |
|  | Heel bone mineral density right | UKBB | 7.41E-09 |
|  | Heel bone mineral density left | UKBB | 1.96E-08 |
| rs3740688 | Arm fat percentage left | UKBB | 1.11E-19 |
|  | Arm fat percentage right | UKBB | 1.03E-17 |
|  | Comparative body size at age 10 | UKBB | 1.27E-16 |
|  | Trunk fat percentage | UKBB | 2.25E-14 |
|  | Body mass index | UKBB | 5.00E-14 |
|  | Arm fat mass left | UKBB | 6.94E-14 |
|  | Arm fat mass right | UKBB | 1.10E-13 |
|  | Body fat percentage | UKBB | 1.36E-13 |
|  | Trunk fat mass | UKBB | 7.65E-12 |
|  | Whole body fat mass | UKBB | 2.27E-11 |
|  | Hip circumference | UKBB | 7.36E-11 |
|  | Leg fat mass left | UKBB | 6.75E-10 |
|  | Waist circumference | UKBB | 1.45E-09 |
|  | Impedance of leg right | UKBB | 4.29E-09 |
|  | Leg fat mass right | UKBB | 4.69E-09 |
|  | Leg fat percentage left | UKBB | 7.04E-09 |
|  | Suffer from nerves | UKBB | 9.73E-09 |
|  | Age at menarche | UKBB | 4.73E-08 |
| rs7412 | APOB apolipoprotein B | 23100282 | 5.90E-154 |
|  | APOB apolipoprotein B response after 40mg daily simvastatin treatment | 23100282 | 4.90E-29 |
|  | Arm fat mass left | UKBB | 2.01E-08 |
|  | Arm fat mass right | UKBB | 5.01E-09 |
|  | Arm fat percentage left | UKBB | 1.83E-08 |
|  | Arm fat percentage right | UKBB | 1.61E-08 |
|  | Body fat percentage | UKBB | 1.13E-09 |
|  | Cholesterol ldl | 22331829 | 2.00E-47 |
|  | Cholesterol total | 25961943 | 8.00E-239 |
|  | Chronic ischaemic heart disease | UKBB | 1.43E-08 |
|  | Coronary artery disease | 29212778 | 7.30E-27 |
|  | HDL cholesterol | 28270201 | 6.00E-14 |
|  | High density lipoprotein | 28334899 | 1.44E-08 |
|  | High light scatter percentage of red cells | 27863252 | 2.95E-38 |
|  | High light scatter reticulocyte count | 27863252 | 9.58E-36 |
|  | High light scatter reticulocyte percentage of red cells | 27863252 | 3.00E-38 |
|  | Hip circumference | UKBB | 2.68E-09 |
|  | Ideal cardiovascular health clinical and behavioural | 27179730 | 9.00E-16 |
|  | Illnesses of father: heart disease | UKBB | 9.03E-09 |
|  | Immature fraction of reticulocytes | 27863252 | 4.36E-38 |
|  | LDL cholesterol | 23100282 | 2.10E-215 |
|  | LDL cholesterol among a group where cancer diabetes hyperhypothyroidism and LDL altering medications were not present | 23067351 | 6.30E-11 |
|  | LDL cholesterol change with statins | 20031582 | 5.54E-30 |
|  | LDL cholesterol levels | 28334899 | 2.00E-286 |
|  | LDL cholesterol response after 40mg daily simvastatin treatment | 23100282 | 2.70E-30 |
|  | LDL cholesterol response to statins baseline LDL cholesterol | 22331829 | 1.60E-47 |
|  | LDL cholesterol response to statins fractional change in LDL cholesterol | 22331829 | 5.80E-19 |
|  | LDL cholesterol response to statins residuals of the measure of fractional change in LDL cholesterol | 22331829 | 4.00E-17 |
|  | Lipid metabolism | 22286219 | 3.00E-58 |
|  | Lipid metabolism phenotypes | 22286219 | 3.00E-58 |
|  | Lipid traits | 24023260 | 2.00E-30 |
|  | Lipoprotein a levels | 28512139 | 3.00E-10 |
|  | Lipoprotein associated phospholipase A2 activity change in response to darapladib treatment in cardiovascular disease | 28753643 | 2.00E-27 |
|  | Lipoprotein associated phospholipase A2 activity Lp2 | 23118302 | 4.30E-81 |
|  | Lipoprotein phospholipase A2 activity in cardiovascular disease | 28753643 | 8.00E-78 |
|  | Lipoproteina levels adjusted for apolipoproteina isoforms | 28512139 | 3.00E-09 |
|  | Low density lipoprotein | 24097068 | 0.00E+00 |
|  | Low density lipoprotein cholesterol | 28548082 | 3.00E-19 |
|  | Medication for cholesterol, blood pressure or diabetes: cholesterol lowering medication | UKBB | 2.85E-62 |
|  | Medication for cholesterol, blood pressure or diabetes: none of the above | UKBB | 4.34E-18 |
|  | Medication for pain relief, constipation, heartburn: aspirin | UKBB | 2.30E-08 |
|  | Metabolite levels lipoprotein measures | 27005778 | 2.00E-120 |
|  | No treatment with medication for cholesterol, blood pressure, diabetes, or take exogenous hormones | UKBB | 1.12E-21 |
|  | Number of treatments or medications taken | UKBB | 2.38E-08 |
|  | Pulse pressure | 28135244 | 4.00E-10 |
|  | Pulse rate | UKBB | 5.50E-17 |
|  | Red cell distribution width | 27863252 | 2.00E-44 |
|  | Response to statin therapy LDL C | 22331829 | 2.00E-47 |
|  | Reticulocyte count | 27863252 | 8.88E-18 |
|  | Reticulocyte fraction of red cells | 27863252 | 4.00E-20 |
|  | Self-reported angina | UKBB | 1.38E-12 |
|  | Self-reported heart attack or myocardial infarction | UKBB | 3.48E-09 |
|  | Self-reported high cholesterol | UKBB | 4.73E-145 |
|  | Total cholesterol | 28887542 | 8.50E-32 |
|  | Total cholesterol levels | 28334899 | 8.00e-315 |
|  | Treatment with aspirin | UKBB | 5.45E-10 |
|  | Treatment with atorvastatin | UKBB | 4.91E-36 |
|  | Treatment with cholesterol lowering medication | UKBB | 2.09E-86 |
|  | Treatment with ezetimibe | UKBB | 1.05E-11 |
|  | Treatment with lipitor 10mg tablet | UKBB | 1.68E-11 |
|  | Treatment with rosuvastatin | UKBB | 1.72E-10 |
|  | Treatment with simvastatin | UKBB | 5.02E-70 |
|  | Triglycerides | 24097068 | 1.15E-28 |
|  | Trunk fat mass | UKBB | 7.95E-11 |
|  | Trunk fat percentage | UKBB | 1.02E-10 |
|  | Vascular or heart problems diagnosed by doctor: angina | UKBB | 1.28E-14 |
|  | Vascular or heart problems diagnosed by doctor: heart attack | UKBB | 1.29E-08 |
|  | Vascular or heart problems diagnosed by doctor: none of the above | UKBB | 1.40E-09 |
|  | Weight | UKBB | 1.25E-08 |
|  | Whole body fat mass | UKBB | 6.13E-10 |
| rs1081105 | Self-reported high cholesterol | UKBB | 2.82E-25 |
|  | Treatment with cholesterol lowering medication | UKBB | 1.93E-12 |
|  | Medication for cholesterol, blood pressure or diabetes: cholesterol lowering medication | UKBB | 1.66E-11 |
|  | Age-related macular degeneration | 26691988 | 1.69E-09 |
|  | Cause of death: unspecified dementia | UKBB | 3.06E-09 |
|  | Treatment with lipitor 10mg tablet | UKBB | 2.99E-08 |
| rs12151021 | Red cell distribution width | 27863252 | 1.73E-08 |
|  | Mean corpuscular hemoglobin concentration | 27863252 | 2.56E-08 |
| rs147711004 | Self-reported high cholesterol | UKBB | 3.30E-14 |
|  | Medication for cholesterol, blood pressure or diabetes: cholesterol lowering medication | UKBB | 2.42E-12 |
|  | Illnesses of mother: none of the above, group 1 | UKBB | 1.09E-08 |
|  | Coronary artery disease | 29212778 | 3.82E-08 |

SNP, single nucleotide polymorphism.

**Table S5. Associations of instrumental variables with secondary traits from PhenoScanner searches for Parkinson's Disease**

| SNP | Trait | PMID | *P* |
| --- | --- | --- | --- |
| rs114138760 | Cause of death: home | UKBB | 1.37E-10 |
| rs35749011 | Dementia with Lewy bodies | 29263008 | 7.00E-10 |
| rs823118 | Sitting height | UKBB | 9.91E-23 |
|  | Height | UKBB | 2.56E-19 |
|  | Whole body fat-free mass | UKBB | 3.70E-18 |
|  | Whole body water mass | UKBB | 1.63E-17 |
|  | Trunk predicted mass | UKBB | 2.22E-17 |
|  | Trunk fat-free mass | UKBB | 3.50E-17 |
|  | Comparative height size at age 10 | UKBB | 5.40E-17 |
|  | Leg fat-free mass right | UKBB | 6.93E-17 |
|  | Leg predicted mass right | UKBB | 1.12E-16 |
|  | Basal metabolic rate | UKBB | 2.58E-16 |
|  | Arm fat-free mass right | UKBB | 7.91E-16 |
|  | Leg fat-free mass left | UKBB | 1.00E-15 |
|  | Leg predicted mass left | UKBB | 1.53E-15 |
|  | Arm predicted mass right | UKBB | 3.26E-15 |
|  | Arm predicted mass left | UKBB | 5.37E-14 |
|  | Arm fat-free mass left | UKBB | 9.97E-14 |
|  | Hand grip strength right | UKBB | 1.40E-12 |
|  | Hand grip strength left | UKBB | 3.57E-11 |
|  | Weight | UKBB | 3.63E-09 |
|  | Hearing difficulty or problems with background noise | UKBB | 8.64E-09 |
| rs1474055 | Trunk fat-free mass | UKBB | 7.21E-10 |
|  | Whole body fat-free mass | UKBB | 7.66E-10 |
|  | Body mass index | 28892062 | 8.53E-10 |
|  | Trunk predicted mass | UKBB | 1.77E-09 |
|  | Whole body water mass | UKBB | 2.89E-09 |
|  | Leg predicted mass right | UKBB | 3.69E-09 |
|  | Leg fat-free mass right | UKBB | 4.27E-09 |
|  | Arm fat-free mass right | UKBB | 8.49E-09 |
|  | Basal metabolic rate | UKBB | 1.10E-08 |
|  | Arm predicted mass right | UKBB | 2.16E-08 |
|  | Leg fat-free mass left | UKBB | 2.76E-08 |
|  | Nap during day | UKBB | 4.08E-08 |
|  | Leg predicted mass left | UKBB | 4.52E-08 |
| rs12497850 | Pulse rate | UKBB | 7.55E-15 |
|  | Height | UKBB | 1.56E-11 |
|  | Impedance of arm left | UKBB | 7.51E-11 |
|  | Sitting height | UKBB | 1.94E-10 |
|  | Sum eosinophil basophil counts | 27863252 | 2.47E-08 |
|  | Impedance of arm right | UKBB | 2.49E-08 |
|  | Impedance of whole body | UKBB | 2.75E-08 |
| rs55961674 | Pulse rate | UKBB | 4.74E-08 |
| rs34025766 | Height | UKBB | 1.01E-141 |
|  | Sitting height | UKBB | 2.54E-80 |
|  | Trunk predicted mass | UKBB | 8.34E-62 |
|  | Trunk fat-free mass | UKBB | 3.59E-61 |
|  | Comparative height size at age 10 | UKBB | 5.36E-57 |
|  | Whole body fat-free mass | UKBB | 6.34E-52 |
|  | Whole body water mass | UKBB | 1.31E-51 |
|  | Arm predicted mass right | UKBB | 1.29E-44 |
|  | Arm fat-free mass right | UKBB | 7.40E-44 |
|  | Forced vital capacity, best measure | UKBB | 3.02E-42 |
|  | Basal metabolic rate | UKBB | 1.48E-41 |
|  | Forced vital capacity | UKBB | 7.41E-41 |
|  | Arm predicted mass left | UKBB | 2.97E-38 |
|  | Arm fat-free mass left | UKBB | 6.95E-38 |
|  | Leg fat-free mass right | UKBB | 7.60E-37 |
|  | Leg predicted mass right | UKBB | 1.02E-36 |
|  | Leg predicted mass left | UKBB | 1.97E-33 |
|  | Leg fat-free mass left | UKBB | 3.05E-33 |
|  | Forced expiratory volume in 1-second, best measure | UKBB | 9.00E-31 |
|  | Forced expiratory volume in 1-second | UKBB | 2.73E-30 |
|  | Forced expiratory volume in 1-second, predicted | UKBB | 6.47E-28 |
|  | Hand grip strength right | UKBB | 5.01E-19 |
|  | Hand grip strength left | UKBB | 7.10E-19 |
|  | Weight | UKBB | 2.11E-13 |
|  | Birth weight | UKBB | 6.47E-12 |
|  | Peak expiratory flow | UKBB | 8.91E-11 |
|  | Leg fat percentage right | UKBB | 1.13E-10 |
|  | Leg fat percentage left | UKBB | 5.22E-10 |
|  | Vascular or heart problems diagnosed by doctor: none of the above | UKBB | 4.46E-09 |
|  | Vascular or heart problems diagnosed by doctor: high blood pressure | UKBB | 1.41E-08 |
|  | Arm fat percentage right | UKBB | 1.55E-08 |
|  | Time spent watching television | UKBB | 4.34E-08 |
| rs1867598 | Mean platelet volume | 27863252 | 2.88E-08 |
| rs11950533 | Height | UKBB | 5.28E-17 |
|  | Forced vital capacity | UKBB | 2.86E-09 |
| rs4140646 | Self-reported malabsorption or coeliac disease | UKBB | 8.10E-72 |
|  | Primary sclerosing cholangitis | 27992413 | 5.56E-32 |
|  | Lymphocyte count | 27863252 | 6.15E-20 |
|  | Intestinal malabsorption | UKBB | 3.72E-19 |
|  | Mean corpuscular hemoglobin | 27863252 | 3.05E-16 |
|  | White blood cell count | 27863252 | 4.28E-16 |
|  | Hemoglobin concentration | 27863252 | 5.15E-16 |
|  | IgA deficiency | 27723758 | 1.04E-12 |
|  | Monocyte count | 27863252 | 1.92E-12 |
|  | Reticulocyte count | 27863252 | 2.05E-12 |
|  | Mean corpuscular volume | 27863252 | 3.31E-12 |
|  | Forced expiratory volume in 1-second | UKBB | 4.31E-12 |
|  | Reticulocyte fraction of red cells | 27863252 | 1.69E-11 |
|  | Forced vital capacity | UKBB | 6.11E-11 |
|  | Hematocrit | 27863252 | 8.62E-11 |
|  | Schizophrenia | 25056061 | 4.03E-10 |
|  | Treatment with insulin product | UKBB | 5.38E-10 |
|  | Self-reported hyperthyroidism or thyrotoxicosis | UKBB | 8.53E-10 |
|  | Medication for pain relief, constipation, heartburn: paracetamol | UKBB | 1.01E-09 |
|  | Forced expiratory volume in 1-second, best measure | UKBB | 1.07E-08 |
|  | Myeloid white cell count | 27863252 | 1.44E-08 |
|  | Sum eosinophil basophil counts | 27863252 | 1.99E-08 |
|  | Red cell distribution width | 27863252 | 2.39E-08 |
|  | Guilty feelings | UKBB | 2.39E-08 |
| rs9261484 | Rheumatoid arthritis | 24390342 | 6.50E-47 |
|  | Eosinophil count | 27863252 | 1.40E-25 |
|  | Sum eosinophil basophil counts | 27863252 | 1.41E-24 |
|  | Self-reported malabsorption or coeliac disease | UKBB | 6.34E-24 |
|  | High light scatter percentage of red cells | 27863252 | 4.50E-21 |
|  | High light scatter reticulocyte count | 27863252 | 1.05E-20 |
|  | Reticulocyte fraction of red cells | 27863252 | 4.10E-17 |
|  | Reticulocyte count | 27863252 | 3.00E-16 |
|  | Immature fraction of reticulocytes | 27863252 | 4.11E-16 |
|  | White blood cell count | 27863252 | 4.26E-16 |
|  | Eosinophil percentage of white cells | 27863252 | 1.30E-14 |
|  | Eosinophil percentage of granulocytes | 27863252 | 1.03E-12 |
|  | Lymphocyte count | 27863252 | 2.46E-12 |
|  | Platelet count | 27863252 | 6.02E-12 |
|  | Neutrophil percentage of granulocytes | 27863252 | 3.10E-11 |
|  | Myeloid white cell count | 27863252 | 1.94E-10 |
|  | Monocyte count | 27863252 | 7.97E-10 |
|  | Granulocyte count | 27863252 | 1.99E-09 |
|  | Intestinal malabsorption | UKBB | 2.92E-09 |
|  | Sum neutrophil eosinophil counts | 27863252 | 3.92E-09 |
|  | Plateletcrit | 27863252 | 3.92E-08 |
| rs112485576 | Rheumatoid arthritis | 24390342 | 1.00E-250 |
|  | Self-reported rheumatoid arthritis | UKBB | 9.04E-122 |
|  | Treatment with methotrexate | UKBB | 3.48E-84 |
|  | Treatment with insulin product | UKBB | 1.70E-76 |
|  | Other rheumatoid arthritis | UKBB | 7.19E-69 |
|  | Started insulin within one year diagnosis of diabetes | UKBB | 4.53E-66 |
|  | Self-reported asthma | UKBB | 5.52E-63 |
|  | Asthma | UKBB | 1.07E-60 |
|  | Self-reported malabsorption or coeliac disease | UKBB | 2.96E-54 |
|  | Self-reported hypothyroidism or myxoedema | UKBB | 4.08E-46 |
|  | Primary sclerosing cholangitis | 27992413 | 1.83E-42 |
|  | Medication for cholesterol, blood pressure or diabetes: insulin | UKBB | 7.77E-41 |
|  | Insulin-dependent diabetes mellitus | UKBB | 1.96E-37 |
|  | Treatment with insulin | UKBB | 2.56E-37 |
|  | Treatment with folic acid product | UKBB | 1.20E-35 |
|  | Wheeze or whistling in the chest in last year | UKBB | 2.37E-33 |
|  | White blood cell count | 27863252 | 1.16E-30 |
|  | Ulcerative colitis | 26192919 | 4.80E-30 |
|  | Sum neutrophil eosinophil counts | 27863252 | 8.65E-30 |
|  | Granulocyte count | 27863252 | 1.04E-29 |
|  | Number of treatments or medications taken | UKBB | 1.43E-29 |
|  | Seropositive rheumatoid arthritis | UKBB | 2.79E-29 |
|  | Self-reported polymyalgia rheumatica | UKBB | 6.16E-29 |
|  | Myeloid white cell count | 27863252 | 8.03E-29 |
|  | Self-reported type 1 diabetes | UKBB | 2.13E-28 |
|  | Neutrophil count | 27863252 | 1.25E-27 |
|  | Taking other prescription medications | UKBB | 1.46E-27 |
|  | Sum basophil neutrophil counts | 27863252 | 2.05E-27 |
|  | Treatment with levothyroxine sodium | UKBB | 3.33E-27 |
|  | Treatment with ventolin 100micrograms inhaler | UKBB | 5.17E-26 |
|  | Diabetes diagnosed by doctor | UKBB | 1.36E-25 |
|  | Long-standing illness, disability or infirmity | UKBB | 1.79E-25 |
|  | Treatment with sulfasalazine | UKBB | 2.16E-25 |
|  | Inflammatory bowel disease | 26192919 | 2.07E-24 |
|  | Intestinal malabsorption | UKBB | 4.49E-22 |
|  | Self-reported diabetes | UKBB | 7.86E-21 |
|  | IgA deficiency | 27723758 | 2.64E-20 |
|  | Nasal polyp | UKBB | 5.80E-19 |
|  | Number of self-reported non-cancer illnesses | UKBB | 8.92E-19 |
|  | Vitamin and mineral supplements: folic acid or folate | UKBB | 9.72E-19 |
|  | Doctor diagnosed asthma | UKBB | 1.75E-18 |
|  | Eye problems or disorders: diabetes related eye disease | UKBB | 7.50E-18 |
|  | Treatment with prednisolone | UKBB | 3.92E-17 |
|  | Eosinophil count | 27863252 | 3.67E-16 |
|  | Sum eosinophil basophil counts | 27863252 | 8.87E-15 |
|  | Overall health rating | UKBB | 1.44E-14 |
|  | Hand grip strength right | UKBB | 4.81E-14 |
|  | Vitamin and mineral supplements: none of the above | UKBB | 5.50E-14 |
|  | Self-reported multiple sclerosis | UKBB | 3.49E-13 |
|  | Granulocyte percentage of myeloid white cells | 27863252 | 8.87E-13 |
|  | Treatment with seretide 50 evohaler | UKBB | 2.24E-12 |
|  | Hand grip strength left | UKBB | 2.65E-11 |
|  | Illnesses of siblings: diabetes | UKBB | 3.02E-11 |
|  | Treatment with hydroxychloroquine | UKBB | 4.75E-11 |
|  | Treatment with leflunomide | UKBB | 7.32E-11 |
|  | Self-reported nasal polyps | UKBB | 2.74E-10 |
|  | Treatment with sulphasalazine | UKBB | 4.21E-10 |
|  | No blood clot, bronchitis, emphysema, asthma, rhinitis, eczema or allergy diagnosed by doctor | UKBB | 2.43E-09 |
|  | Other disorders of pancreatic internal secretion | UKBB | 2.56E-09 |
|  | Monocyte percentage of white cells | 27863252 | 4.00E-09 |
|  | Treatment with thyroxine product | UKBB | 7.23E-09 |
|  | Forced vital capacity | UKBB | 1.09E-08 |
|  | Medication for pain relief, constipation, heartburn: none of the above | UKBB | 2.37E-08 |
|  | Self-reported eczema or dermatitis | UKBB | 2.39E-08 |
|  | Self-reported ulcerative colitis | UKBB | 2.54E-08 |
|  | Sitting height | UKBB | 4.01E-08 |
|  | Neutrophil percentage of white cells | 27863252 | 4.06E-08 |
|  | Self-reported sarcoidosis | UKBB | 4.42E-08 |
| rs1293298 | Heel bone mineral density | UKBB | 1.41E-10 |
|  | Impedance of leg left | UKBB | 1.88E-10 |
|  | Impedance of leg right | UKBB | 3.19E-09 |
|  | Heel bone mineral density left | UKBB | 4.89E-08 |
| rs72840788 | Height | UKBB | 1.85E-10 |
|  | Sitting height | UKBB | 9.14E-10 |
|  | Pulse rate | UKBB | 3.19E-08 |
| rs10847864 | Mean platelet volume | 27863252 | 2.88E-20 |
| rs9568188 | Alcohol intake frequency | UKBB | 3.30E-08 |
| rs12147950 | Mean corpuscular volume | 27863252 | 9.18E-09 |
|  | Mean corpuscular hemoglobin | 27863252 | 2.27E-08 |
| rs2904880 | Trunk fat mass | UKBB | 3.76E-37 |
|  | Arm fat percentage left | UKBB | 6.09E-35 |
|  | Trunk fat percentage | UKBB | 9.46E-35 |
|  | Hip circumference | UKBB | 2.13E-34 |
|  | Whole body fat mass | UKBB | 1.32E-33 |
|  | Arm fat percentage right | UKBB | 3.27E-33 |
|  | Arm fat mass left | UKBB | 6.94E-33 |
|  | Waist circumference | UKBB | 2.88E-32 |
|  | Body fat percentage | UKBB | 1.06E-31 |
|  | Arm fat mass right | UKBB | 4.04E-31 |
|  | Weight | UKBB | 6.11E-31 |
|  | Leg fat mass left | UKBB | 2.61E-26 |
|  | Leg fat mass right | UKBB | 1.86E-24 |
|  | Leg predicted mass left | UKBB | 3.19E-21 |
|  | Leg predicted mass right | UKBB | 4.82E-21 |
|  | Leg fat-free mass right | UKBB | 4.85E-21 |
|  | Leg fat-free mass left | UKBB | 7.23E-21 |
|  | Leg fat percentage left | UKBB | 1.19E-19 |
|  | Basal metabolic rate | UKBB | 2.81E-17 |
|  | Leg fat percentage right | UKBB | 1.45E-16 |
|  | Waist circumference | 25673412 | 2.70E-13 |
|  | Whole body fat-free mass | UKBB | 2.44E-12 |
|  | Alcohol intake frequency | UKBB | 2.63E-12 |
|  | Whole body water mass | UKBB | 5.08E-12 |
|  | Qualifications: college or university degree | UKBB | 9.29E-11 |
|  | Body mass index adjusted for smoking | 28443625 | 1.50E-10 |
|  | Hip circumference in females | 25673412 | 1.80E-10 |
|  | Comparative body size at age 10 | UKBB | 2.89E-10 |
|  | Intelligence multi trait analysis | 29326435 | 3.00E-10 |
|  | Arm predicted mass left | UKBB | 3.15E-10 |
|  | Arm fat-free mass left | UKBB | 5.39E-10 |
|  | Arm fat-free mass right | UKBB | 3.22E-09 |
|  | Comparative height size at age 10 | UKBB | 4.89E-09 |
|  | Mean corpuscular volume | 27863252 | 5.75E-09 |
|  | Arm predicted mass right | UKBB | 7.39E-09 |
|  | Impedance of leg right | UKBB | 1.17E-08 |
|  | Body mass index | 23001569 | 1.47E-08 |
|  | Body mass index in non-smokers | 28443625 | 1.52E-08 |
|  | Waist circumference in males | 25673412 | 2.00E-08 |
|  | Types of physical activity in last 4 weeks: light diy | UKBB | 2.31E-08 |
|  | Body mass index adjusted for physical activity | 28448500 | 4.86E-08 |
| rs6500328 | Crohns disease | 26192919 | 1.48E-23 |
|  | Inflammatory bowel disease | 26192919 | 1.58E-11 |
| rs12600861 | Height | UKBB | 1.01E-47 |
|  | Sitting height | UKBB | 4.38E-35 |
|  | Trunk fat-free mass | UKBB | 1.32E-20 |
|  | Trunk predicted mass | UKBB | 2.52E-20 |
|  | Whole body fat-free mass | UKBB | 5.33E-15 |
|  | Whole body water mass | UKBB | 1.62E-14 |
|  | Arm fat-free mass left | UKBB | 1.07E-13 |
|  | Arm fat-free mass right | UKBB | 1.22E-13 |
|  | Arm predicted mass left | UKBB | 3.67E-13 |
|  | Arm predicted mass right | UKBB | 9.08E-13 |
|  | Basal metabolic rate | UKBB | 2.88E-11 |
|  | Comparative height size at age 10 | UKBB | 1.26E-08 |
|  | Arm fat percentage left | UKBB | 3.10E-08 |
| rs12951632 | Arm fat percentage right | UKBB | 1.74E-11 |
|  | Arm fat percentage left | UKBB | 5.58E-11 |
|  | Arm fat mass left | UKBB | 2.82E-10 |
|  | Arm fat mass right | UKBB | 2.98E-10 |
|  | Waist circumference | UKBB | 6.58E-09 |
|  | Trunk fat mass | UKBB | 8.79E-09 |
|  | Body fat percentage | UKBB | 1.25E-08 |
|  | Body mass index | UKBB | 1.27E-08 |
|  | Trunk fat percentage | UKBB | 1.74E-08 |
|  | Whole body fat mass | UKBB | 2.19E-08 |
| rs2269906 | Reticulocyte fraction of red cells | 27863252 | 2.30E-28 |
|  | Reticulocyte count | 27863252 | 5.64E-26 |
|  | Mean corpuscular hemoglobin | 27863252 | 7.77E-20 |
|  | Red cell distribution width | 27863252 | 4.19E-18 |
|  | Trunk fat percentage | UKBB | 2.18E-13 |
|  | Body fat percentage | UKBB | 5.52E-13 |
|  | Arm fat percentage left | UKBB | 2.75E-11 |
|  | Arm fat percentage right | UKBB | 4.44E-11 |
|  | Trunk fat mass | UKBB | 2.52E-10 |
|  | Whole body fat mass | UKBB | 4.77E-10 |
|  | Leg fat percentage right | UKBB | 7.19E-10 |
|  | Leg fat percentage left | UKBB | 5.18E-09 |
|  | Immature fraction of reticulocytes | 27863252 | 5.60E-09 |
|  | Sitting height | UKBB | 5.75E-09 |
|  | Mean corpuscular hemoglobin concentration | 28017375 | 1.00E-08 |
|  | Leg fat mass right | UKBB | 2.06E-08 |
|  | Leg fat mass left | UKBB | 3.36E-08 |
|  | Arm fat mass right | UKBB | 3.56E-08 |
|  | Arm fat mass left | UKBB | 4.86E-08 |
| rs850738 | Mean platelet volume | 27863252 | 4.22E-33 |
|  | Platelet count | 27863252 | 2.31E-17 |
|  | Platelet distribution width | 27863252 | 1.19E-13 |
| rs62053943 | Hair or balding pattern: pattern 4 | UKBB | 1.09E-36 |
|  | Nap during day | UKBB | 9.50E-24 |
|  | Red blood cell count | 27863252 | 2.60E-23 |
|  | Red cell distribution width | 27863252 | 3.08E-20 |
|  | Forced vital capacity | UKBB | 6.77E-18 |
|  | Hematocrit | 27863252 | 1.02E-17 |
|  | Relative age of first facial hair | UKBB | 1.89E-17 |
|  | Forced vital capacity, best measure | UKBB | 8.09E-17 |
|  | Eosinophil percentage of granulocytes | 27863252 | 9.00E-17 |
|  | Sitting height | UKBB | 1.48E-16 |
|  | Hemoglobin concentration | 27863252 | 3.76E-16 |
|  | Forced expiratory volume in 1-second | UKBB | 4.85E-16 |
|  | Hair or balding pattern: pattern 3 | UKBB | 1.17E-15 |
|  | Eosinophil percentage of white cells | 27863252 | 1.81E-15 |
|  | Neutrophil percentage of granulocytes | 27863252 | 2.35E-15 |
|  | Sensitivity or hurt feelings | UKBB | 3.93E-15 |
|  | Forced expiratory volume in 1-second, best measure | UKBB | 2.36E-14 |
|  | Pain type experienced in last month: headache | UKBB | 6.34E-13 |
|  | Neuroticism score | UKBB | 2.73E-12 |
|  | Impedance of whole body | UKBB | 4.80E-12 |
|  | Eosinophil count | 27863252 | 9.89E-12 |
|  | Reticulocyte count | 27863252 | 2.72E-11 |
|  | Impedance of arm left | UKBB | 2.89E-11 |
|  | Medication for pain relief, constipation, heartburn: paracetamol | UKBB | 5.97E-11 |
|  | Forced expiratory volume in 1-second, predicted percentage | UKBB | 4.31E-10 |
|  | Treatment with paracetamol | UKBB | 6.43E-10 |
|  | Qualifications: college or university degree | UKBB | 6.94E-10 |
|  | High light scatter reticulocyte count | 27863252 | 7.54E-10 |
|  | Sum eosinophil basophil counts | 27863252 | 8.12E-10 |
|  | Impedance of arm right | UKBB | 1.33E-09 |
|  | Mood swings | UKBB | 1.61E-09 |
|  | Relative age voice broke | UKBB | 2.91E-09 |
|  | High grade serous ovarian cancer | 28346442 | 3.11E-09 |
|  | Neutrophil percentage of white cells | 27863252 | 4.30E-09 |
|  | Invasive ovarian cancer | 28346442 | 5.02E-09 |
|  | Mouth or teeth dental problems: mouth ulcers | UKBB | 8.84E-09 |
|  | Serous invasive ovarian cancer | 28346442 | 1.36E-08 |
|  | Miserableness | UKBB | 3.49E-08 |
|  | Systolic blood pressure | UKBB | 4.01E-08 |
| rs117615688 | Forced expiratory volume in 1-second | UKBB | 4.42E-15 |
|  | Forced vital capacity | UKBB | 6.06E-15 |
|  | Forced vital capacity, best measure | UKBB | 5.44E-14 |
|  | Hair or balding pattern: pattern 4 | UKBB | 8.01E-14 |
|  | Forced expiratory volume in 1-second, best measure | UKBB | 1.87E-13 |
|  | Sitting height | UKBB | 3.63E-12 |
|  | Red blood cell count | 27863252 | 2.06E-11 |
|  | Hematocrit | 27863252 | 9.02E-10 |
|  | Relative age of first facial hair | UKBB | 1.67E-09 |
|  | Hemoglobin concentration | 27863252 | 4.71E-09 |
|  | Neuroticism score | UKBB | 8.52E-09 |
| rs11658976 | Red cell distribution width | 27863252 | 7.36E-09 |

SNP, single nucleotide polymorphism.

**Table S6.** Results of MR analysis of the causal relationship between two common neurodegenerative diseases and stroke after exclusion of potentially pleiotropic SNPs.

| Method | Alzheimer's disease | | | | Parkinson's Disease | | | |
| --- | --- | --- | --- | --- | --- | --- | --- | --- |
|  | SNPs (N) | *OR* | 95%*CI* | P-value | SNPs (N) | *OR* | 95%*CI* | P-value |
| Stroke |  |  |  |  |  |  |  |  |
| IVW | 11 | 1.00 | 0.95~1.06 | 0.956 | 60 | **1.05** | **1.02~1.08** | **0.001** |
| Weighted median | 11 | 0.97 | 0.91~1.05 | 0.496 | 60 | 1.04 | 0.99~1.09 | 0.085 |
| MR Egger | 11 | / | / | 0.137^a^ | 60 | / | / | 0.432^a^ |
| MR-PRESSO | 11 | / | / | 0.405^b^ | 60 | / | / | 0.864^b^ |
| Ischemic stroke |  |  |  |  |  |  |  |  |
| IVW | 11 | 0.98 | 0.92~1.03 | 0.413 | 60 | **1.05** | **1.02~1.09** | **0.001** |
| Weighted median | 11 | 0.96 | 0.89~1.04 | 0.313 | 60 | **1.05** | **1.00~1.11** | **0.042** |
| MR Egger | 11 | / | / | 0.251^a^ | 60 | / | / | 0.376^a^ |
| MR-PRESSO | 11 | / | / | 0.377^b^ | 60 | / | / | 0.533^b^ |
| Intracerebral hemorrhage |  |  |  |  |  |  |  |  |
| IVW | 4 | 1.08 | 0.55~2.14 | 0.817 | 47 | 1.01 | 0.82~1.24 | 0.947 |
| Weighted median | 4 | 0.97 | 0.51~1.86 | 0.929 | 47 | 1.04 | 0.76~1.42 | 0.807 |
| MR Egger | 4 | / | / | 0.679^a^ | 47 | / | / | 0.139^a^ |
| MR-PRESSO | 4 | / | / | 0.217^b^ | 47 | / | / | 0.679^b^ |

SNP, single nucleotide polymorphism; OR, odds ratio; CI, confidence interval; IVW, Inverse-variance-weighted; MR, Mendelian randomization; MR-PRESSO, MR Pleiotropy RESidual Sum and Outlier.

^a^*P*-value of the intercept from MR Egger regression analysis.

^b^*P*-value of MR-PRESSO global test

**
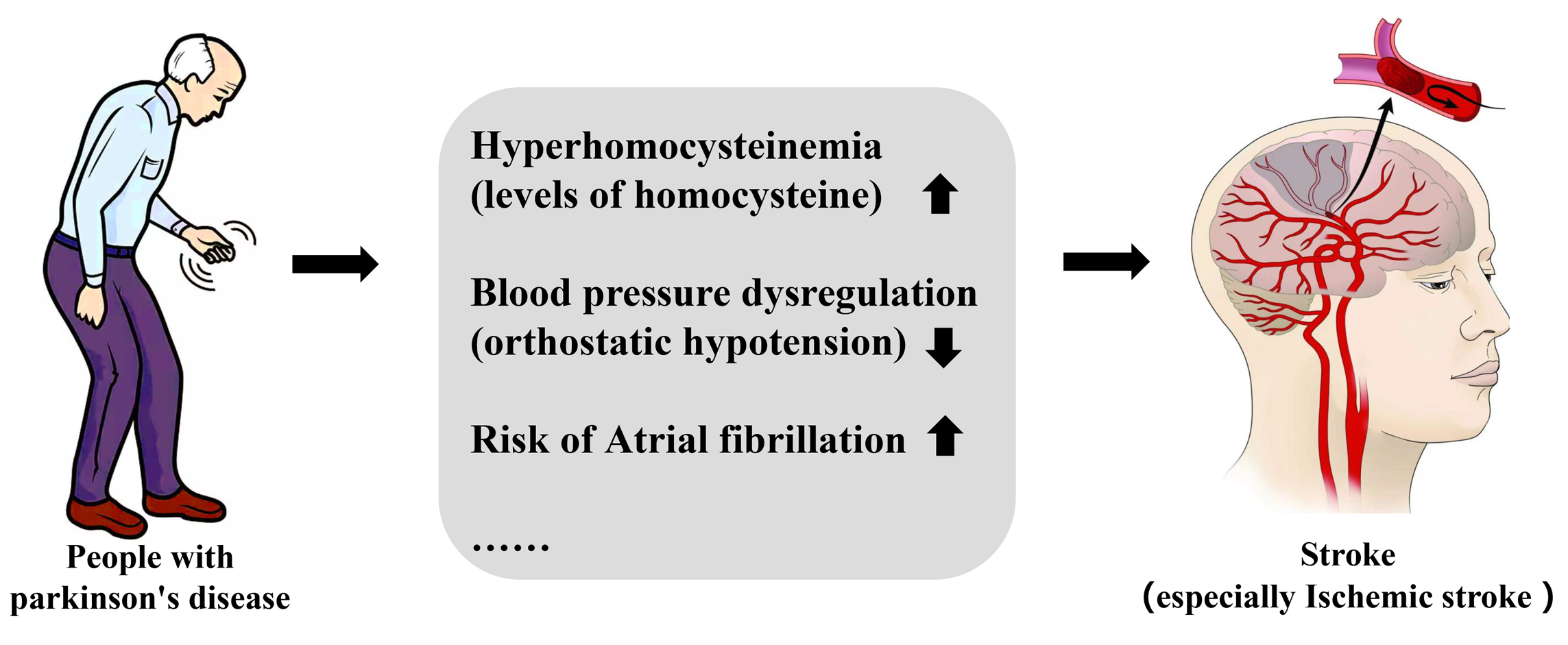
**

**Figure S1.** The mechanisms underlying the causal relationship between parkinson's disease and stroke risk

**Details of MEGASTROKE consortium members**

We gratefully acknowledge the contributing studies and databases (MEGASTROKE project) that made GWAS summary data available. We acknowledge the contributions of Malik R, Chauhan G, Traylor M, Sargurupremraj M, Okada Y, Mishra A, Rutten-Jacobs L, Giese AK, van der Laan SW, Gretarsdottir S, Anderson CD, Chong M, Adams HHH, Ago T, Almgren P, Amouyel P, Ay H, Bartz TM, Benavente OR, Bevan S, Boncoraglio GB, Brown RD Jr, Butterworth AS, Carrera C, Carty CL, Chasman DI, Chen WM, Cole JW, Correa A, Cotlarciuc I, Cruchaga C, Danesh J, de Bakker PIW, DeStefano AL, den Hoed M, Duan Q, Engelter ST, Falcone GJ, Gottesman RF, Grewal RP, Gudnason V, Gustafsson S, Haessler J, Harris TB, Hassan A, Havulinna AS, Heckbert SR, Holliday EG, Howard G, Hsu FC, Hyacinth HI, Ikram MA, Ingelsson E, Irvin MR, Jian X, Jiménez-Conde J, Johnson JA, Jukema JW, Kanai M, Keene KL, Kissela BM, Kleindorfer DO, Kooperberg C, Kubo M, Lange LA, Langefeld CD, Langenberg C, Launer LJ, Lee JM, Lemmens R, Leys D, Lewis CM, Lin WY, Lindgren AG, Lorentzen E, Magnusson PK, Maguire J, Manichaikul A, McArdle PF, Meschia JF, Mitchell BD, Mosley TH, Nalls MA, Ninomiya T, O'Donnell MJ, Psaty BM, Pulit SL, Rannikmäe K, Reiner AP, Rexrode KM, Rice K, Rich SS, Ridker PM, Rost NS, Rothwell PM, Rotter JI, Rundek T, Sacco RL, Sakaue S, Sale MM, Salomaa V, Sapkota BR, Schmidt R, Schmidt CO, Schminke U, Sharma P, Slowik A, Sudlow CLM, Tanislav C, Tatlisumak T, Taylor KD, Thijs VNS, Thorleifsson G, Thorsteinsdottir U, Tiedt S, Trompet S, Tzourio C, van Duijn CM, Walters M, Wareham NJ, Wassertheil-Smoller S, Wilson JG, Wiggins KL, Yang Q, Yusuf S, Amin N, Aparicio HS, Arnett DK, Attia J, Beiser AS, Berr C, Buring JE, Bustamante M, Caso V, Cheng YC, Choi SH, Chowhan A, Cullell N, Dartigues JF, Delavaran H, Delgado P, Dörr M, Engström G, Ford I, Gurpreet WS, Hamsten A, Heitsch L, Hozawa A, Ibanez L, Ilinca A, Ingelsson M, Iwasaki M, Jackson RD, Jood K, Jousilahti P, Kaffashian S, Kalra L, Kamouchi M, Kitazono T, Kjartansson O, Kloss M, Koudstaal PJ, Krupinski J, Labovitz DL, Laurie CC, Levi CR, Li L, Lind L, Lindgren CM, Lioutas V, Liu YM, Lopez OL, Makoto H, Martinez-Majander N, Matsuda K, Minegishi N, Montaner J, Morris AP, Muiño E, Müller-Nurasyid M, Norrving B, Ogishima S, Parati EA, Peddareddygari LR, Pedersen NL, Pera J, Perola M, Pezzini A, Pileggi S, Rabionet R, Riba-Llena I, Ribasés M, Romero JR, Roquer J, Rudd AG, Sarin AP, Sarju R, Sarnowski C, Sasaki M, Satizabal CL, Satoh M, Sattar N, Sawada N, Sibolt G, Sigurdsson Á, Smith A, Sobue K, Soriano-Tárraga C, Stanne T, Stine OC, Stott DJ, Strauch K, Takai T, Tanaka H, Tanno K, Teumer A, Tomppo L, Torres-Aguila NP, Touze E, Tsugane S, Uitterlinden AG, Valdimarsson EM, van der Lee SJ, Völzke H, Wakai K, Weir D, Williams SR, Wolfe CDA, Wong Q, Xu H, Yamaji T, Sanghera DK, Melander O, Jern C, Strbian D, Fernandez-Cadenas I, Longstreth WT Jr, Rolfs A, Hata J, Woo D, Rosand J, Pare G, Hopewell JC, Saleheen D, Stefansson K, Worrall BB, Kittner SJ, Seshadri S, Fornage M, Markus HS, Howson JMM, Kamatani Y, Debette S, Dichgans M
